# An Information-Centric Semantic Data Collection Tree for Wireless Sensor Networks

**DOI:** 10.3390/s20216168

**Published:** 2020-10-29

**Authors:** Ngoc-Thanh Dinh, Younghan Kim

**Affiliations:** School of Electronic Engineering, Soongsil University, Room 1104, Huyngam Engineering Building 424, Sangdo-dong, Dongjak-Gu, Seoul 06978, Korea; younghak@dcn.ssu.ac.kr

**Keywords:** internet of things, IoT service function chains, network function virtualization, fog computing, edge computing, high availability, J0101

## Abstract

Data collection is an important application of wireless sensor networks (WSNs) and Internet of Things (IoT). Current routing and addressing operations in WSNs are based on IP addresses, while data collection and data queries are normally information-centric. The current IP-based approach incurs significant management overheads and is inefficient for semantic data collection and queries. To address the above issue, this paper proposes a semantic data collection tree (sDCT) construction scheme to build up a semantic data collection tree for wireless sensor networks. The semantic tree is rooted at the edge/sink and supports data collection tasks, queries, and configurations efficiently. We implement the sDCT in Contiki and evaluate the performance of the sDCT in comparison with the state-of-the-art scheme, 6LoWPAN/RPL and L2RMR, using telosb sensors under various scenarios. The obtained results show that the sDCT achieves a significant improvement in terms of the energy efficiency and the packet transmissions required for data collection or a query task compared to 6LoWPAN/RPL and L2RMR.

## 1. Introduction

Data collection and data queries are important applications in wireless sensor networks (WSNs) and Internet of Things (IoT). For that reason, a data collection tree is normally built to support both periodic data collection and query-based data collection for a group of sensor nodes. The current standardized data collection tree for WSNs is built based on 6LoWPAN [1] and the corresponding routing protocol, RPL [2]. We call the current scheme the IP-based data collection tree (ipDCT). In the IP-based data collection tree, the data collection is built from the edge, or in other words, the sink node. The tree is built to support traffic patterns required for data collection and data queries, including point-to-multi-point (P2MP), multi-point-to-point (MP2P), and point-to-point (P2P) traffic patterns. An example of a P2MP traffic pattern is when the edge sends a request to all temperature sensors for data collection or configuration. An example of an MP2P traffic pattern is when all sensors or a group of sensors report their data to the edge. All operations for packet forwarding in the tree to support those traffic patterns are based on IP addresses. However, the data collection and data queries in WSNs are normally information-centric [3,4,5]. Users are interested in sensing data in an area, not addresses of sensors. For example, a user requests temperature data from all temperature sensors in an area’s WSN. The mismatching between the user semantic query model and the data collection tree building approach incurs complicated processes required for resource-constrained sensor nodes at both the network layer and the application layer.

In the IP-based data collection tree, to build the routing paths [6] for MP2P traffic, a DIO (DODAG Information Object) message is used to construct a Directed Acyclic Graph (DAG) [2]. The DIO message is broadcasted from the sink node downward to the child nodes. Each node receives DIO messages with DODAG configuration information to configure themselves and selects parent nodes based on the ranking to be reachable from the DODAG root. By this way, the DAG is constructed for MP2P traffic flows. To build the routing paths for P2MP, the DODAG root triggers a Destination Advertisement (DA) mechanism in the DIO and the traffic flows outward, from the sink to other nodes. DA timers are configured such that DAs start at greater depth. In this process, each node sends a Destination Advertisement Object (DAO) message with its route information upward to the edge/sink node. The routing information has to be stored in the sink nodes and upward nodes along the path to the sink node. With a non-storing node, the source routing is used. By this way, DAs build up the routing state to support outward P2MP traffic flows, from the sink to other nodes [2,7]. For P2P traffic [8], when two arbitrary routers need to communicate, the data packets are restricted to travel only along with the links in the DAG. This requires the pre-establishment of routes for each potential destination. The DAG is built to optimize the routing path to the sink, so restricting P2P routes to use only the DAG links may result in many sub-optimal routes. Additional procedures are then added for P2MP and P2P traffic. Each traffic type requires a different construction and initialization, which incurs a high overhead for routing states and route discovery, which are fairly heavy with resource-constrained nodes. To support semantic data collection and data queries, extra node discovery processes in the application layer are also required, which are inefficient for resource-constrained sensors.

For the same purpose of supporting semantic data collection and data queries, this paper proposes a semantic data collection tree construction scheme for the edge to build up a semantic data collection tree (sDCT) for wireless sensor networks based on information-centric networking [4,9,10,11]. The scheme facilitates forwarding operations of traffic patterns for common data collection and data queries in WSNs. We observe that a WSN is a special type of network where each sensor produces a special type of sensing data (e.g., a temperature sensor produces only temperature sensing data). This does not like nodes in the Internet, where nodes can produce various types of data. Based on the observation, we propose a semantic naming scheme for sensor nodes as well as their content objects [3]. We then design a semantic and lightweight data collection tree construction scheme starting from the edge. The proposed semantic data collection tree (sDCT) supports common traffic patterns in WSNs natively without requiring route lookup or completed route information. In particular, the sDCT supports multiple levels for multicast packet forwarding without requiring extra communication overhead and multicast group member management. We implemented the sDCT in Contiki and evaluated the performance of the sDCT in comparison with ipDCT and L2RMR using telosb sensors under various scenarios. The obtained results show that the sDCT achieves a significant improvement in terms of the energy efficiency and the packet transmissions required for data collection or a query task compared to ipDCT and L2RMR [12].

## 2. Related Work

**Figure 1:**
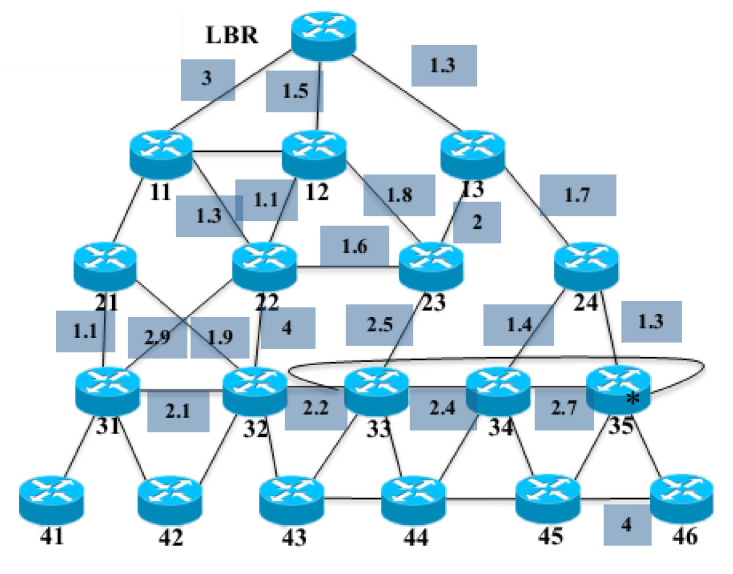
An illustration of ranking and route building for nodes in a routing protocol (RPL)/6LoWPAN network (LBR is the edge border router).

The current standardized data collection tree for WSNs is built based on 6LoWPAN [1] and the corresponding routing protocol, RPL [2], which is quite complicated and experiences a high overhead [13,14,15]. RPL/6LoWPAN builds the tree topology as follows. RPL builds a Directed Acyclic Graph (DAG) and uses the DAG to direct the computation of routing paths. In particular, an Object Function (OF) is in conjunction with constraints and routing metrics to calculate the ranking of nodes in the DAG. The rank of a node in RPL is the relative position of the node with respect to other nodes in the network. Figure 1 illustrates the ranking of nodes of a network in the RPL.

The routing path computation of the RPL for different traffic patterns is quite complicated. For different traffic patterns, the RPL uses different types of messages and path discovery/setup operations to build routing states for the routing paths [2]. For the MP2P traffic pattern, the RPL defines DIO messages, which are broadcasted from the edge to the child nodes. Upon receiving the DIO messages, each sensor configures itself to join the network. For the P2MP traffic pattern, the RPL implements the Destination Advertisement (DA) mechanism using Destination Advertisement Object (DAO) messages for the outward traffic flows. For that, each sensor transmits DAO messages with its routing information toward edge so that the DA mechanism is then run to build up routing states for P2MP traffic patterns. For the P2P traffic, the RPL requires nodes to pre-establish routing paths for every potential destination. The RPL results in a high overhead for each P2P traffic flow. A similar approach is also used for discovering routes to multiple targets (multicast), which creates a DAG rooted at the source node and connected to multicast members.

For multicasting, RPL implements MPL, an IPv6 multicast forwarding protocol adapted for communication of resource-constrained devices like sensors [7]. The multicast protocol requires different control messages, seeds, seed identifiers, and communication overhead to establish and maintain routes for multicast transmission. The main idea of designing RPL using a DAG is to optimize routing cost to the DAG’s root for MP2P traffic. Additional procedures are then added for P2MP and P2P traffic. Each traffic type requires a different construction and initialization. However, this results in a high overhead for routing states and route discovery, which are fairly heavy with constrained nodes. The design may be necessary for communication in all cases. However, this incurs a high overhead for simple data collection and data queries in wireless sensor networks. In WSNs, the main communication patterns are quite simple, as follows. The edge sends configuration or data query requests to one or a group of nodes in the network. Nodes in the network periodically send their sensing data or report their sensing data upon requests to the edge. It is important to note that the data collection and data queries are normally executed in an information-centric manner. For example, a request is sent to query sensing data from sensors belonging to a type or from sensors in a subnetwork. This paper proposes a simple information-centric semantic data collection tree to address this issue.

The Collection Tree Protocol CTP [16] is also a well-known collection tree protocol in wireless sensor networks. The study [17,18] shows advantages of the RPL in terms of improving the packet reception ratio and energy consumption in comparison with CTP. Other surveys of routing protocols in WSNs can also be found in [19,20,21]. In this paper, we focus on the high-level operations and initial ideas for data collection in WSNs. Therefore, in the evaluation part, we compare the sDCT with RPL/6LoWPAN (ipDCT) under fair configurations of the sDCT in comparison with ipDCT.

Information-centric networking (ICN) [22,23,24,25] is considered a promising approach for Internet of Things [26,27,28]. Recently, a number of works have been investigated to explore the benefits of ICN for IoT [10,11,29,30,31,32]. The literature review for information-centric IoT is presented in [10,11]. However, there are still many challenges that need to be addressed to apply ICN to IoT [33,34]. This paper addresses a unique issue of data collection trees in WSNs with ICN.

## 3. The Proposed Semantic Data Collection Tree

### 3.1. Semantic Naming Scheme

The proposed semantic naming structure for sensor nodes and their data objects includes two parts [3]. The first part expresses a real-world category name of the sensor, which is called the category prefix (CP). The latter part is the ID of the node, which makes the name persistent and unique. We utilize a semantic name that allows direct verification based on binding the associated information object, the category of information, and the name. For example, a temperature sensor may have a name of “temp::1325”, in which “temp” is the category prefix indicating a temperature sensor and “::1325" is the ID of the node. We present a mechanism to construct the ID in the next section. The name can be a flat name or a hierarchical name. This paper describes the naming scheme using a flat style.

To build up the semantic data collection tree, we use the sensor category prefix as the prefix of a name. We define category prefixes for different types of sensors; for example, “temp” for temperature sensors, “ligh” for light sensors, “humi” for humidity sensors, etc. For simplicity, we assume that there is a standardized list of sensor types. The category prefix is then exploited for semantic data collection and data queries. Note that for a fair comparison with the IP-based data collection tree using 6LoWPAN and RPL, we implement the semantic data collection tree using a 128-bit name length, which is the same as the length of an Ipv6 address. However, in the illustration using a small network with a limited number of nodes and a limited number of sensor types, we use only 16 bits for the category prefix and 16 bits for the ID. The rest of the name length is illustrated using “::”. This means that only a 32-bit address is required for the experimental network.

### 3.2. ID Construction

This subsection describes how the sDCT constructs the IDs for sensor nodes. The IDs are designed not only to make nodes unique, but also to facilitate routing operations in WSNs. For the ID construction, we modify the node rank discovery in the RPL to construct an informational node rank. In the RPL, the node rank discovery is designed for loop avoidance only. In the sDCT, the node rank is used as the ID of the node, which contains information about how packets can be forwarded to the node. In other words, the rank also contains routing information to reach the node.

The function for generating the ranking-based IDs in sDCT is comparable with RPL routing. The rank of a node in sDCT also expresses the position relative to other nodes; however, this is in a defined order for easy coordination among nodes. Nodes form a ranked tree topology, which is similar to the multibit-tries [35] for router table indexing. The purpose of the ranking-based ID is to make routing become easier and more efficient by minimizing the communication for route discovery from a source node to a destination. We define a maximum number of children for a node as 15, similarly to RPL, for a fair comparison. Each node manages the ordered and joined slots and the number of child nodes in order to allocate the ranks of the child nodes. A node allocates an ordered slot for only one child node at a time. Each node joins the network in an ordered slot corresponding with an ordered slot number and a parent ID obtained from its parent node. Because the parent ID is unique and each child node receives a different ordered slot, thus, the generated ID is also unique.

The ID configuration is executed from the edge downward to the child nodes. The edge node initiates a unique ID automatically by itself, or it assigned by a mechanism to guarantee that its ID is unique among sink nodes in the networks (for example, ::0001). The rank of the *i*th child node, $R_{i\mathrm{th}}$, is calculated as follows.

(1) $$R_{i\mathrm{th}}=R_{parent}\;*\;16+HEX\left[i\;\mathrm{mod}\;15\right].$$

**Figure 2:**
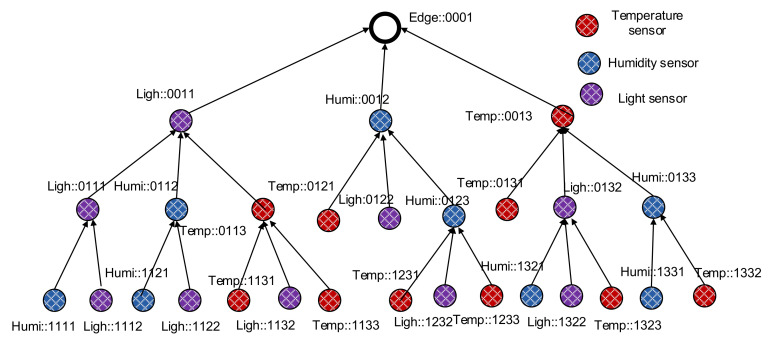
A semantic data collection construction starting from the edge node downward to the child nodes.

For example, if the edge node has the ID “::0001” ($R_{parent}$ is “::0001”), its child nodes will have the ID range from “::0011” to “::001F”. Similarly, if a node has the ID “::0011” ($R_{parent}$ is “::0011”), its child nodes will have the ID range from “::0111” to “::011F”. When a new node N turns on, it sends a rank discovery message to its one hop neighbors. Neighbor nodes receiving the discovery message respond with their rank and the ordered slot of N if they have available slots of additional child nodes. The new node N then selects the nearest node as its parent and calculates its rank as its ID. The new node then creates its full name from the ID and sends a verification message to the parent node for verifying and registering. After verifying a child node, the parent adds the new node to its neighbor table with the face ID in which it receives the verification message from the new node. The process is executed continuously until all nodes are ranked and participate in the ranked tree network; an example is shown in Figure 2.

Figure 2 illustrates an example of a semantic data collection tree constructed starting from the edge node downward to child nodes. The same of each node shows its semantic information, including its type of sensor and its rank in the tree. With this semantic data collection tree design, data queries for specific types of sensing data can be facilitated.

**Figure 3:**
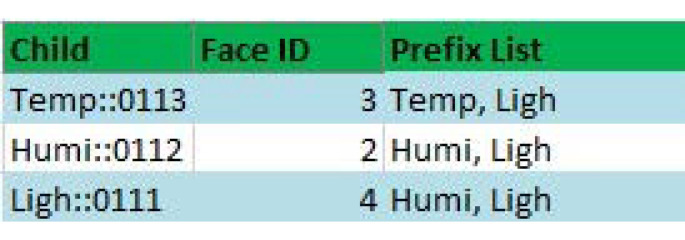
An example of a semantic child table of node Ligh::001. The table contains a list of child nodes, their face ID, and the list of prefixes under their subtree.

During the rank discovery process, each node builds up a table containing child nodes’ information, similarly to the RPL. However, the child table in sDCT contains the child nodes’ name, face ID, and a list of prefixes existing under the subtree of the child nodes, as shown in Figure 3. The table helps a node know which types of sensors are available under its subtree. In other words, a node knows which types of sensing data that the node or its child nodes can provide. This information is later used for semantic multicast forwarding. For example, if the edge wants to send a packet to temperature sensors, then the packet is forwarded to only subtrees that have the prefix of “Temp”. As a result, a sensing data query is forwarded to a subnetwork only if the subnetwork produces the matching type of sensing data. The design is to improve the efficiency of packet forwarding inside a WSN, thus saving energy for sensors.

### 3.3. Packet Format and Traffic Patterns

**Figure 4:**
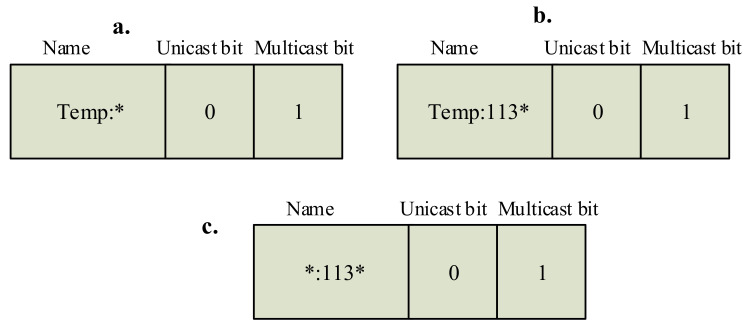
Each interest packet header consists of the name of interest, the unicast bit, and the multicast bit. The semantic data collection tree (sDCT) supports multiple levels of multicast: (**a**) a type of sensor in a network, (**b**) a type of sensor in a subnetwork and (**c**) to sensors in a subnetwork.

Each interest packet header in sDCT consists of the name of interest, a unicast bit, and a multicast bit, as shown in Figure 4. The name in the interest packet expresses the type of data of interest (i.e., temperature data or light data, etc.) and the level of the network of interest (i.e., in the whole network or only in a subnetwork). For example, Figure 4a illustrates an interest packet for the temperature data (i.e., temp) in the whole network level (i.e., ::*). Figure 4b illustrates an interest packet for the temperature data (i.e., temp) in the subnetwork (i.e., ::113*). Figure 4c illustrates an interest packet for any sensing data (i.e., *) produced by the subnetwork (i.e., ::113*).

The unicast bit and the multicast bit are added to enable the sDCT to support different traffic patterns that are common in WSNs and supported by 6LoWPAN/RPL. When only the unicast bit turns on, the packet is forwarded as a unicast message and the full name is used to forward the packet to a specific node. For illustration, an interest packet with the name “temp::1131”, the unicast bit = 1, and the multicast bit = 0 will be forwarded as a unicast message to the node “temp::1131”. When both the unicast bit and multicast bit turn on to 1, the packet is propagated as a broadcast packet. For illustration, an interest packet with the name “*::113*”, the unicast bit = 1, and the multicast bit = 1 will be forwarded as a broadcast message to all nodes in the subnetwork “::113*”. When both the unicast bit and multicast bit are 0, the packet is forwarded as an anycast packet. For illustration, an interest packet with the name “temp::113*”, the unicast bit = 0, and the multicast bit = 0 will be forwarded as an anycast message to any temperature node in the subnetwork “::113*”. When only the multicast bit turns on, the packet is forwarded as a multicast packet. For illustration, an interest packet with the name “temp::113*”, the unicast bit = 0, and the multicast bit = 1 will be forwarded as all temperature sensors in the subnetwork “::113*”.

By design, the sDCT natively supports different levels of multicast traffic, including (1) multicast to all nodes belonging to a type of node in the network (i.e., temperature sensors), (2) multicast to all nodes belonging to a type of node in a subtree, and (3) multicast to all nodes in a subtree. The levels are determined based on the name of interest. Under a multicast mode (i.e., multicast bit = 1), if the name contains only the category prefix (e.g., ID is 0000), the packet is sent via multicast to all nodes belonging to that category prefix. For illustration, if the name “temp::0000” is used, the packet is then sent via multicast to all temperature sensors. If the name “temp:113*” is used, the packet is sent via multicast to all temperature sensors under the subnetwork with ID “113*”. For that, the packet is first sent via unicast to the subnetwork with ID “113*” and then sent via multicast to all temperature nodes in the subnetwork with ID “113*”. If the name “*:113*” is used without a prefix, the packet is sent via multicast to all nodes under the subnetwork with ID “113*”. For that, the packet is first sent via unicast to the subnetwork with ID “113*” and then sent via multicast to all nodes in the subnetwork with ID “113*”.

### 3.4. Packet Forwarding for Different Types of Traffic Patterns

#### 3.4.1. MP2P

**Figure 5:**
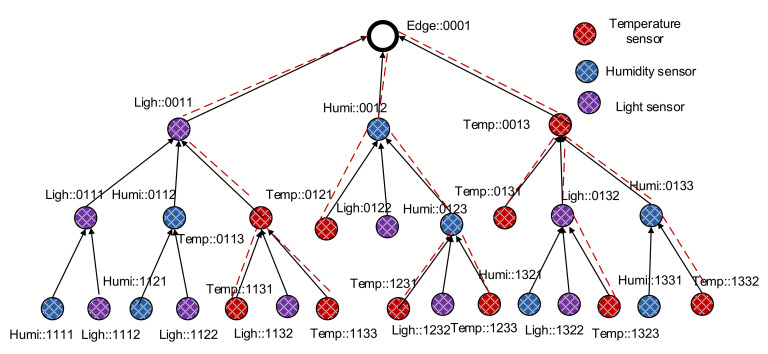
An illustration for multi-point-to-point (MP2P) from temperature sensors to the edge in the sDCT.

For the MP2P, the packet forwarding in the sDCT is similar to that in ipDCT, where nodes forward their sensing data to the parent nodes toward the edge. However, the sDCT can support semantic data aggregation natively to reduce the number of packet transmissions. For example, temperature data of temperature sensors can be aggregated together along the forwarding path without requiring a semantic classification implemented with a complicated aggregation scheme, as illustrated in Figure 5.

#### 3.4.2. P2P or Unicast Forwarding

A unicast packet is forwarded by parsing the ID in the destination name of interest. In the sDCT, the unicast packet forwarding does not require route lookup or completed route information. The reason is that the name ID of the destination reflects the path to reach the destination already. Therefore, the unicast packet forwarding is processed based on parsing the ID of the name and comparing with the ID of the current node. For illustration, if the Edge::0001 in Figure 5 wants to send a packet to the node “temp::1131” with unicast bit = 1 and multicast bit = 0, the edge then parses the ID “1131” and compares with its ID “1000” using the longest matching. The edge then forwards the packet to the next hop with ID “11**”, that is, the ID of the node “Ligh::0011”. At the node Ligh::0011, the node parses the ID “1131” and compares with its ID “0011”. The node then forwards the packet to the next hop “113*”, which is the ID of the node “Temp::0113”. At the node “Temp::0113”, the node parses the ID “1131” and compares with its ID “0113”. The packet is then forwarded to the destination “temp::1131”. In this way, the unicast packet is forwarded without requiring route storage and route lookup. This helps save significant resources and energy consumption for resource-constrained sensors. Moreover, this enables the sDCT to support light-weight or non-storage nodes.

#### 3.4.3. P2MP

**Figure 6:**
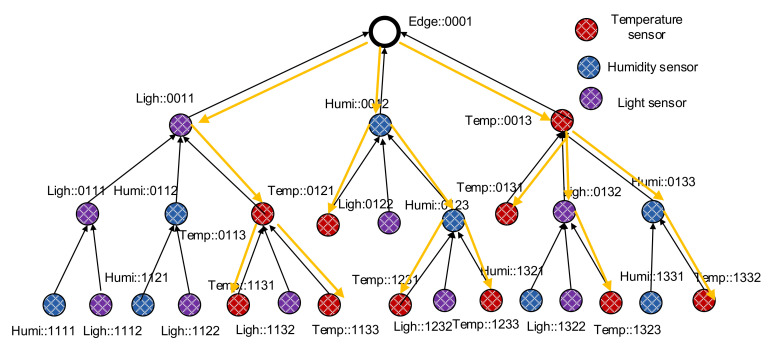
An illustration for multicast packet forwarding in the sDCT. The multicast packet from the edge is forwarded to all temperature sensors in the sDCT.

P2MP traffic is treated using multicast forwarding in the sDCT. The sDCT supports native multicast naturally without requiring multicast member registration and member management, as in IP multicast. Following the construction of the sDCT, nodes know which types of sensors exist in their child nodes’ subtrees. As a result, semantic P2MP packet forwarding is facilitated. For illustration, when the edge sends a multicast packet to all temperature sensors in its network (e.g., using the name of interest “Temp::*” with unicast bit = 0 and multicast bit = 1). The packet is forwarded downstream based on the list of prefixes. A node forwards the multicast packet only to the child nodes that have the “Temp” prefix in their subtree networks. The packet is then forwarded continuously to all temperature sensors in the network, as shown in Figure 6.

## 4. Performance Evaluation

**Figure 7:**
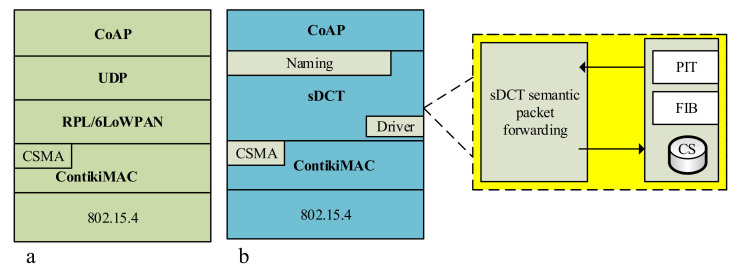
Implementation stack of the sDCT (**b**) in comparison with the ipDCT (**a**).

We implement a prototype of the sDCT in Contiki [36], as shown in Figure 7, in comparison with the existing RPL/6LoWPAN ipDCT and L2RMR [12], the state-of-the-art studies. Figure 7a shows the protocol stack of a RPL/6LoWPAN node and its improved version, L2RMR. Figure 7b shows the protocol stack of a sDCT node.

We present the performance evaluation of the proposed sDCT with experimental and analysis results compared with the state-of-the-art ipDCT and L2RMR. We perform extensive evaluations for the sDCT in comparison with ipDCT and L2RMR using the Cooja simulator available in Contiki [37]. The simulations consist of an edge node with 20 temperature sensors, 20 light sensors, and 20 humidity sensors. The name prefixes of sensors are pre-configured. The detailed configurations for simulations are presented in Table 1. We use the default packet length of RPL/6LoWPAN for the sDCT and L2RMR. For the radio noise model, we use the closest-fit-pattern matching (CPM) [38]. We implement counters to track changes and record the time in each radio state of sensors to measure the duty cycle of a node [39]. We set the CCA (Clear Channel Assessment) check parameter up to 400 times. Other parameters are kept the same as the default configurations of the Cooja CC2420 radio model [38].

### 4.1. Bootstrapping

**Figure 8:**
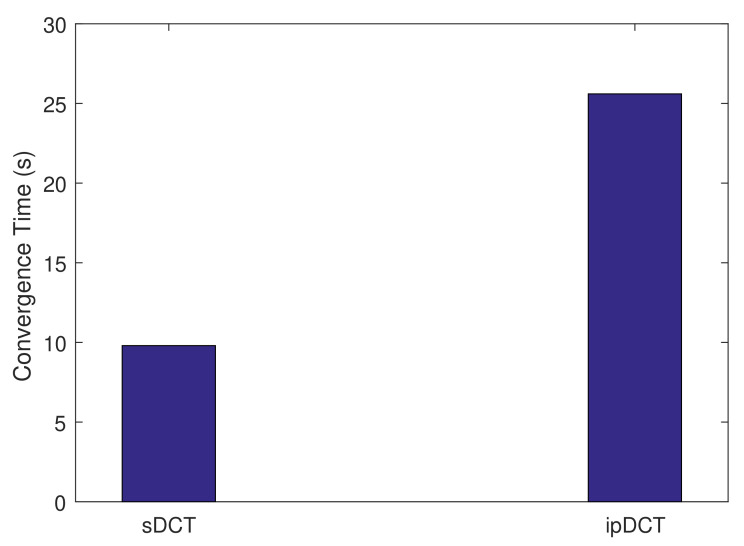
Convergence time for the network setup.

We first evaluate the sDCT, L2RMR, and ipDCT during the bootstrapping (network setup). We measure the convergence time of the network setup. The obtained results are presented in Figure 8. Figure 8 shows that the collection tree setup in the sDCT is significantly faster than in ipDCT and L2RMR. This is due to the fact that ipDCT and L2RMR use a high number of control messages, such as DIO, DAO, and DIS (DODAG Informational Solicitation) messages, during the network setup and maintenance. The rank building in the sDCT is also simpler than in ipDCT and L2RMR. L2RMR requires more message exchanges for the bootstrapping, so its convergence time is longer than that of ipDCT.

**Figure 9:**
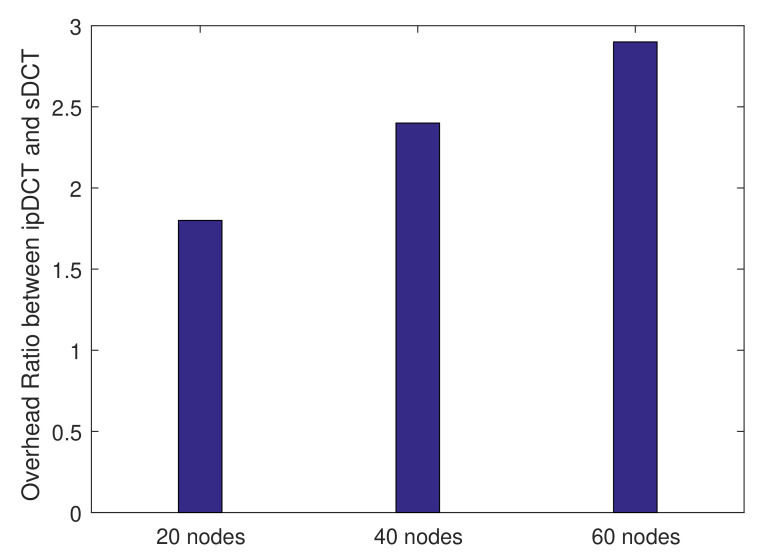
Overhead ratio between sDCT and RPL/6LoWPAN (ipDCT) for the network setup under different numbers of nodes (the number of messages is counted as the overhead).

We count the number of messages used during the bootstrapping of the two protocols (sDCT and ipDCT). The number of messages is used as the transmission overhead of the sDCT and ipDCT during bootstrapping. We then compare the overhead ratio between ipDCT and sDCT under different numbers of network nodes. The obtained results are presented in Figure 9. Figure 9 shows that ipDCT experiences a significantly higher transmission overhead than the sDCT. The reasons are explained in the previous results. Figure 9 also indicates that the higher the number of nodes in the network that we obtain, the greater the overhead ratio between ipDCT and sDCT. Note that the results of L2RMR are worse than those of ipDCT, as explained in the previous figure, so we do not compare the results of L2RMR with those of the sDCT.

### 4.2. P2MP Traffic

**Figure 10:**
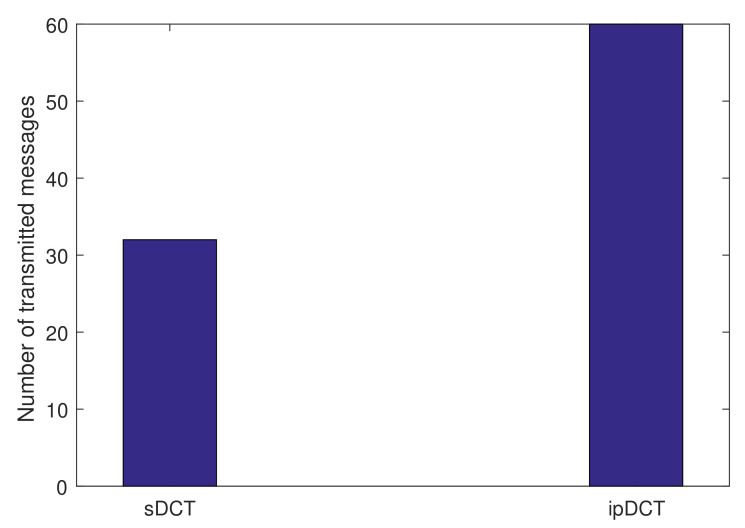
Point-to-multi-point (P2MP) traffic overhead comparison between sDCT, L2RMR, and ipDCT.

We now execute experiments with P2MP traffic in the sDCT, L2RMR, and ipDCT. In particular, the edge is assigned a task to send configuration messages to all temperature sensors. We count the number of transmitted messages required for the edge to complete its task as the task overhead. Figure 10 presents the number of transmitted messages required for the edge to send a configuration message to all temperature sensors. The figure shows that the sDCT requires only 32 messages while the ipDCT requires about 60 messages. L2RMR and RPL have the same results, which means that L2RMR has no improvement in terms of P2MP traffic in comparison with ipDCT. In particular, sDCT requires the message transmission only at related nodes, including temperature sensors and intermediate nodes, while the task overhead of ipDCT is similar to broadcasting. The obtained result shows that the sDCT is more efficient in terms of energy and transmission overhead than ipDCT in the cases of semantic requests/queries or configuration.

### 4.3. MP2P Traffic

**Figure 11:**
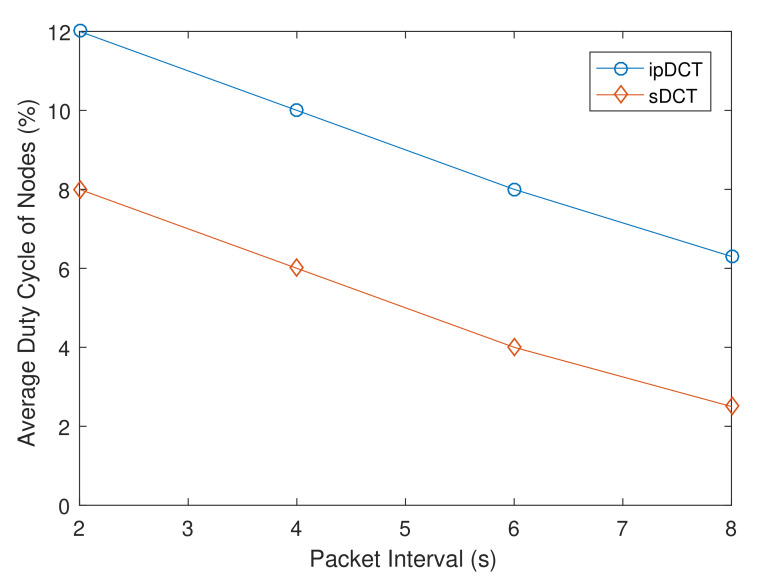
MP2P traffic overhead comparison between sDCT, L2RMR, and ipDCT.

For MP2P traffic experiments, every node is required to report its sensing data to the edge in every packet interval. We vary the packet interval from 2 to 8 s. We then measure the average duty cycle of sensors in the sDCT, L2RMR, and ipDCT. The obtained results are presented in Figure 11. Figure 11 shows that the sDCT achieves the lowest average duty cycle of nodes. L2RMR achieves a significantly lower average duty cycle of nodes compared to ipDCT, but the result is higher than that of the sDCT. Their number of data messages is quite similar. However, ipDCT and L2RMR require frequent control messages to maintain the tree.

### 4.4. P2P Traffic

**Figure 12:**
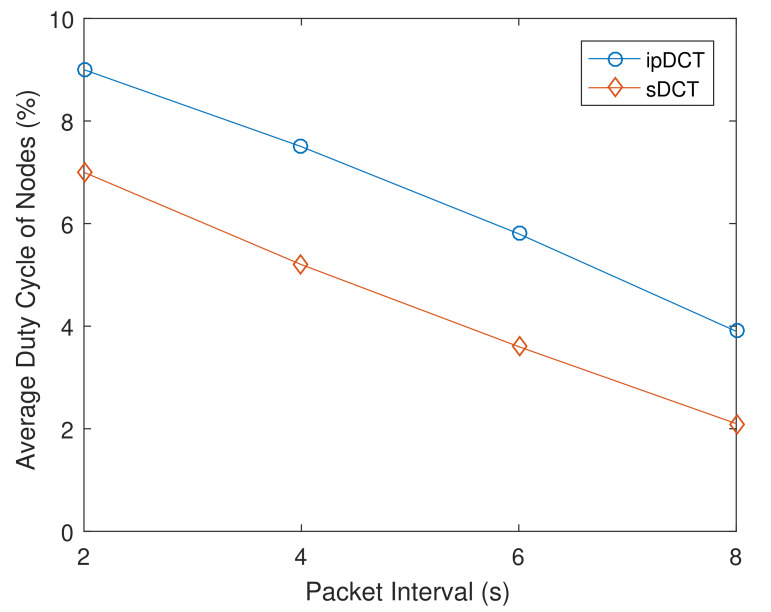
Point-to-point (P2P) traffic overhead comparison between sDCT, L2RMR, and ipDCT.

For P2P traffic experiments, the network randomly selects 20 pairs of nodes to communicate P2P under various packet intervals from 2 to 8 s. The obtained average duty cycle results of nodes in the sDCT, L2RMR, and ipDCT are presented in Figure 12. Figure 12 shows that the sDCT is more energy efficient than ipDCT and L2RMR in cases of P2P traffic patterns. The result is that L2RMR and ipDCT require more control messages to establish the P2P routes. L2RMR achieves a slight improvement in terms of the average duty cycle of nodes compared to ipDCT in the P2P scenario.

## 5. Discussion and Conclusions

This paper presents a simple design to build the semantic data collection tree for wireless sensor networks based on an information-centric approach. The data collection tree building approach matches with the approach for sensing data queries and data collection. Therefore, the sDCT facilitates all types of traffic patterns in WSNs. The operations required to support each type of traffic patterns are minimal and natural based on the semantic tree. Through extensive evaluation, the sDCT has shown its advantages and efficiency over ipDCT in terms of energy efficiency and packet transmissions. This work just focuses on a high-level network comparison, while protocols at the lower network level are kept the same as those of the ipDCT stack. In future works, we plan to explore cross-layer approaches for the sDCT and discover how the information-centric approach can benefit the packet transmissions in WSNs at the lower layer. We move toward building a completed semantic protocol stack for WSNs based on the sDCT and designing a simple way to build applications on the top of the stack.

## Figures and Tables

**Table 1:** Parameters.

Parameter	Value	Parameter	Value
CCA check parameter	400 times	channel sampling	10 ms
transmission range	50 m	simulation time	60 min
maximum number of child nodes	15	Noise model	CPM
confidence interval	95 %	packet size	127 octets
